# p53 Restoration in Induction and Maintenance of Senescence: Differential Effects in Premalignant and Malignant Tumor Cells

**DOI:** 10.1128/MCB.00747-15

**Published:** 2016-01-19

**Authors:** Mohamad Harajly, Hasan Zalzali, Zafar Nawaz, Sandra E. Ghayad, Farah Ghamloush, Hussein Basma, Samiha Zainedin, Wissam Rabeh, Mark Jabbour, Ayman Tawil, Danielle A. Badro, Gerard I. Evan, Raya Saab

**Affiliations:** a Department of Pediatric and Adolescent Medicine, American University of Beirut Medical Center, Beirut, Lebanon; b Cytogenetics and Molecular Cytogenetic Laboratory, Hamad General Hospital, Doha, Qatar; c Department of Pathology and Laboratory Medicine, American University of Beirut Medical Center, Beirut, Lebanon; d Department of Biochemistry, University of Cambridge, Cambridge, United Kingdom

## Abstract

The restoration of p53 has been suggested as a therapeutic approach in tumors. However, the timing of p53 restoration in relation to its efficacy during tumor progression still is unclear. We now show that the restoration of p53 in murine premalignant proliferating pineal lesions resulted in cellular senescence, while p53 restoration in invasive pineal tumors did not. The effectiveness of p53 restoration was not dependent on p19^Arf^ expression but showed an inverse correlation with Mdm2 expression. In tumor cells, p53 restoration became effective when paired with either DNA-damaging therapy or with nutlin, an inhibitor of p53-Mdm2 interaction. Interestingly, the inactivation of p53 after senescence resulted in reentry into the cell cycle and rapid tumor progression. The evaluation of a panel of human supratentorial primitive neuroectodermal tumors (sPNET) showed low activity of the p53 pathway. Together, these data suggest that the restoration of the p53 pathway has different effects in premalignant versus invasive pineal tumors, and that p53 activation needs to be continually sustained, as reversion from senescence occurs rapidly with aggressive tumor growth when p53 is lost again. Finally, p53 restoration approaches may be worth exploring in sPNET, where the p53 gene is intact but the pathway is inactive in the majority of examined tumors.

## INTRODUCTION

Cellular senescence is defined as irreversible cell cycle exit induced by tumor-promoting insults, such as oncogene expression, DNA damage, telomere attrition, or loss of tumor suppressors (1). The irreversibility of the senescent state has been suggested because senescent cells are resistant to mitogenic stimulation and do not reenter the cell cycle when exposed to conditions that stimulate proliferation in quiescent cells. Senescent cells have been found in premalignant tumors, and senescence is thought to contribute to tumor suppression by leading to cell cycle exit in premalignant lesions that have undergone a primary tumorigenic insult and/or mutation (2–4). However, the irreversibility of the senescent state *in vivo* has been questioned, since by definition a proportion of premalignant lesions progress to invasive tumors even after long periods of time, suggesting either that few cells have not undergone true senescence and are able to revert to the cell cycle or that senescent cells resume proliferation if exposed to further genetic insults affecting key pathways that are relevant to the maintenance of cell cycle exit (4).

The p53 tumor suppressor protein has been well established to be central to the induction of cellular senescence in most systems studied (5). In addition, the RB protein also plays a central role and is essential for senescence induction in most contexts (6). The roles of these two proteins in the maintenance of senescence have been studied primarily in the setting of replicative senescence, which is a cell culture phenomenon driven by telomere attrition in cultured cells (6). In replicative senescence, dual inactivation of p53 and RB seems to be sufficient for the reversion of cells into the cell cycle, while the abolishment of components of the p53 pathway alone or of the RB pathway alone were found to have various effects in different cell types, on cell cycle entry, on cell division versus crisis, and on the ability to proliferate (7–9). Importantly, the ability of cells to actually divide and survive cell division varied among these studies, with some cell lines primarily undergoing crisis and cell death, while others were able to survive and proliferate. As opposed to replicative senescence, few studies have evaluated the stability of oncogene-induced and DNA damage-induced senescence (7, 10, 11), the states that are most relevant to tumor suppression in hyperplastic premalignant lesions *in vivo*. These studies have focused primarily on cell culture systems, using mouse and human fibroblasts, and showed differing efficacy on reversion of RAS- and RAF-induced senescence using different cell types.

In the current study, we evaluated the effect of p53 restoration on premalignant and malignant tumors using a mouse model of cyclin D1-driven pineoblastoma. We also assessed the stability of oncogene-induced cellular senescence by dissecting the role of p53 in its maintenance using both *in vitro* and *in vivo* models.

## MATERIALS AND METHODS

### Mouse studies.

*Irbp-Cyclin D1* transgenic mice (12) were bred with *p53*^−/−^ mice (Jackson Laboratory, ME) or *p53ERTAM*^*ki/ki*^ mice (10) and maintained in a mixed C57BL/6 × 129/Sv genetic background. Animals were euthanized at defined time points or when obviously ill, in accordance with the American University of Beirut Institutional Animal Care and Use Committee guidelines; all animal studies were approved by this committee. Tamoxifen (Sigma-Aldrich) was administered as an intraperitoneal injection of 1 mg once daily (10, 13–15). Irradiation was given at 2.5 Gy from a [Cs]^137^ source (10).

### Cell culture and viral transduction.

Pineal cells were explanted by plating onto 8-well Permanox chamber slides (Nunc, Rochester, NY) and cultured in Dulbecco's modified Eagle's medium (DMEM) with 10% fetal bovine serum (FBS) and a mixture of 1% glutamine and 1% penicillin-streptomycin (1% Pen-Strep). Medium was renewed every 3 days. Cells were treated with 100 nM 4-hydroxytamoxifen (4OHT), 4 μM nutlin (Abcam Biochemicals), or 10 μM etoposide as specified. Mouse embryonic fibroblasts (MEFs) were isolated from embryonic day 13.5 (E13.5) embryos and cultured in DMEM containing 10% fetal bovine serum, 1% nonessential amino acids, 1% sodium pyruvate, 1% glutamine, and 1% Pen-Strep. Retrovirus was produced using a p*MSCV-RasV12-IRES-GFP* expression plasmid (Clontech), with virus production via calcium phosphate transfection in HEK 293T cells with the appropriate packaging plasmids, as in our previous studies (13). Virus was added to cells with 8 μg/ml Polybrene (hexadimethrine bromide; Sigma). Spinoculation was performed at 32°C with 2,500 rpm for 2 h, and medium was replaced after 3 h. The following day, the procedure was repeated.

### SABG, BrdU, and TUNEL assays.

Senescence-associated beta-galactosidase (SABG) staining was performed as described in our previous studies (16). The quantification of positive cells in pineal primary cultures was done using the color range tool in Adobe Photoshop CS6. Positive (blue) and negative (pink) cells were chosen using the Eyedropper tool, and the number of blue and pink pixels was assessed using the histogram tool. For MEFs, SABG-positive cells were counted manually and normalized to the total cell number. For bromodeoxyuridine (BrdU) incorporation assays, cells were treated with BrdU at a concentration of 33 μM for 2 h, fixed with 50% methanol–50% acetone solution, treated with 2N HCl, neutralized by borate buffer, probed with anti-BrdU (Santa Cruz Biotechnology) antibody, and then detected using cyanine 3 secondary antibody (Jackson ImmunoResearch Laboratories). Terminal deoxynucleotidyltransferase-mediated dUTP-biotin nick end labeling (TUNEL) assay was performed using the *in situ* cell death kit, fluorescein (Roche, Mannheim, Germany), according to the manufacturer's instructions. Stained cells were mounted with aqueous medium containing 4′,6-diamidino-2-phenylindole (DAPI; Vector Laboratories, Burlingame, CA), placed under coverslips, and visualized by immunofluorescence microscopy. For quantitation, stained cells were manually counted from at least 5 representative fields, at ×40 magnification, and normalized to the total cell numbers, which were counted as DAPI-positive nuclei.

### Cell accumulation and colony formation assays.

For cell accumulation assays, *p53ERTAM*^*Ki*/−^ MEFs were plated in 6-well plates at a density of 200,000 cells per well, transduced with *RasV12* retrovirus as described above, and treated with 4OHT to restore p53. 4OHT treatment then was continued or withdrawn depending on the condition, and 7 days later cells were fixed with 3:1 methanol-acetic acid and stained with 0.5% cresyl violet in methanol. For the soft-agar colony formation assay, 0.8% base layer SeaPlaque agarose (Lonza) in 1.5 ml DMEM containing 10% fetal bovine serum, 1% nonessential amino acids, 1% sodium pyruvate, 1% glutamine, and 1% Pen-Strep was plated onto each well of 6-well plates, and cells suspended in 0.48% SeaPlaque agarose (Lonza) were added to the agar. Medium was replaced every 3 days. The plates were incubated at 37°C in 5% CO_2_, and cells were treated as described above for the accumulation assay. Two weeks later the clones were counted and photographed.

### Histological studies.

Tissue was fixed in 4% paraformaldehyde for 72 h and then embedded in paraffin. Four- to 8-μm sections were deparaffinized, stained with hematoxylin and eosin, or incubated with anti-Ki67 (BD-Pharmingen, San Diego, CA), anti-DEC1, anti-p15^Ink4b^, anti-p21^Cip1^, anti-p14^Arf^, anti-MDM2, anti-p19Arf (all from Santa Cruz Biotechnology), or anti-pERK (Cell signaling). They then were detected using a Vectastain kit (Vector Laboratories) and DAB substrate kit (Dako, Carpinteria, CA) per the manufacturers' instructions. For immunofluorescence staining, anti-H3K9me3 (Upstate Laboratories, Syracuse, NY) antibody was detected with Alexa Fluor 488 secondary antibodies (Jackson ImmunoResearch Laboratories) and counterstained with DAPI (Vector Laboratories). Antigen retrieval was performed in a steamer in citrate antigen retrieval buffer (pH 6.0). Apoptosis was detected using a TUNEL *in situ* cell death kit (Roche, Mannheim, Germany) according to the manufacturer's instructions. Fluorescent *in situ* hybridization (FISH) for a 17p deletion with the Vysis Inc.LSI p53 probe at 17p13.1 was performed by standard clinical testing at the cytogenetics laboratory at the American University of Beirut Medical Center.

### p53 gene mutation analysis.

DNA was extracted from paraffin-embedded tissue using phenol-chloroform followed by 3 M sodium acetate and ethanol precipitation. *p53* exons 4 to 11 were amplified using the following primers: exon 4 forward (f), 5′-CTGAGGACCTGGTCCTCTGACTG-3′; reverse (r), 5′-AGGCATTGAAGTCTCATGGAA-3′; exon 5 f, 5′-CTCCTGAGGTGTAGACGCCAACTCTCTCTA-3′; r, 5′-TGGGCAACCAGCCCTGTCGTCTCTCCA-3′; exon 6 f, 5′-CGATGGTGAGCAGCTGGGGCTGGA-3′; r, 5′-TAGGGAGGTCAAATAAGCAGCAGGAGAAAG-3′; exon 7 f, 5′-AAAAAAAAAAAAAAGGCCTCCCCTG-3′; r, 5′-GGATGGGTAGTAGTATGGAAG-3′; exon 8 f, 5′-TCCTGGAGCTGGAGCTTAGGC-3′; r, 5′-AAGTGAATCTGAGGCATAACTGCAC-3′; exon 9 f, 5′-GTTATGCCTCAGATTCACT-3′; r, 5′-TGAGTGTTAGACTGGAAACT-3′; exon 10 f, 5′-ACTTACTTCTCCCCCTCCTCTGTTGCTGC-3′; r, 5′-ATGGAATCCTATGGCTTTCCAACCTAGGAAG-3′; exon 11 f, 5′-CTCACTCATGTGATGTCATCTC-3′; r, 5′-CTGACGCACACCTATTGCAA-3′. Amplicons were separated by agarose electrophoresis, extracted, and purified using a QIAquick PCR purification kit (Qiagen) and then sequenced using an Avant genetic analyzer AB3130A machine. The NCBI databank (http://www.ncbi.nlm.nih.gov/BLAST) was used to check for mutations and polymorphisms.

### Western blotting.

Cells were lysed in universal lysis buffer (16). Protein was quantified using the Bradford assay and normalized prior to loading. Electrophoresis was performed using 12% Tris-chloride gels and transferred to polyvinylidene difluoride membranes (Bio-Rad Laboratories, Hercules, CA), blocked with 5% nonfat milk in TBST, probed using antibodies to Dec1, p21^Cip1^, p16^Ink4a^, p15^Ink4b^, glyceraldehyde-3-phosphate dehydrogenase (GAPDH) (all from Santa Cruz Biotechnology, Santa Cruz, CA), p53 (Novocastra), and phospho-p53 (Cell Signaling), and detected using horseradish peroxidase (HRP)-conjugated secondary antibodies (all from Santa Cruz Biotechnology, Santa Cruz, CA) using ECL detection reagent (Roche).

### Quantitative real-time PCR.

Total RNA was extracted using TRIzol reagent (Ambion) according to the manufacturer's instructions and treated with DNase I (Qiagen). cDNA was synthesized using a RevertAid first-strand cDNA synthesis kit (Fermentas). Real-time PCR was done with the iQ SYBR green supermix kit in a CFX96 system (Bio-Rad Laboratories). Amplification was performed using the following primers: *GAPDH* sense, AGCCAAAAGGGTCATCATCT; antisense, GGGGCCATCCACAGTCTTCT; p19Arf sense, GCCGCACCGGAATCCT; antisense, TTGAGCAGAAGAGCTGCTACGT. PCR conditions included denaturation at 95°C for 15 min, 40 cycles of 95°C for 15 s, 72°C for 1 min, and then annealing at 55°C.

### Bisulfite modification and methylation-specific PCR.

Bisulfite conversion was conducted using the EpiTect bisulfite kit (Qiagen) according to the manufacturer's instructions. Bisulfite-treated DNA was amplified by primers specific for either methylated (sense, 5′-GTGTTAAAGGGCGGCGTAGC-3′; antisense, 5′-AAAACCCTCACTCGCGACGA-3′) or unmethylated (sense, 5′-TTTTTGGTGTTAAAGGGTGGTGTAGT-3′; antisense, 5′-CACAAAAACCCTCACTCACAACAA-3′) sequences, as previously described (17). PCR was carried out using MeltDoctor HRM master mix (Applied Biosystems) at 95°C for 10 min (enzyme activation), followed by 39 cycles of 95°C for 30 s, 60°C for 30 s, and 72°C for 1 min and a final elongation at 72°C for 10 min. All reaction mixtures were run with unmethylated and methylated human bisulfite-treated DNA as controls (Epitect PCR control DNA set; Qiagen). The PCR products were separated by electrophoresis through a 1.5% agarose gel containing ethidium bromide. DNA bands were visualized by UV light.

### Statistical and image analysis.

Comparisons between experimental groups were performed using Student's *t* test, and a *P* value below 0.05 was considered statistically significant. Digital photomicrographs were obtained using an LSM 710 confocal laser-scanning microscope (Carl Zeiss, Germany). Composite images were constructed using Photoshop CS6 software (Adobe Systems, Mountain View, CA).

## RESULTS

### p53 restoration induces senescence in proliferating premalignant pineal cells *in vitro* and in premalignant hyperplastic pineal lesions *in vivo*.

**FIG 1:**
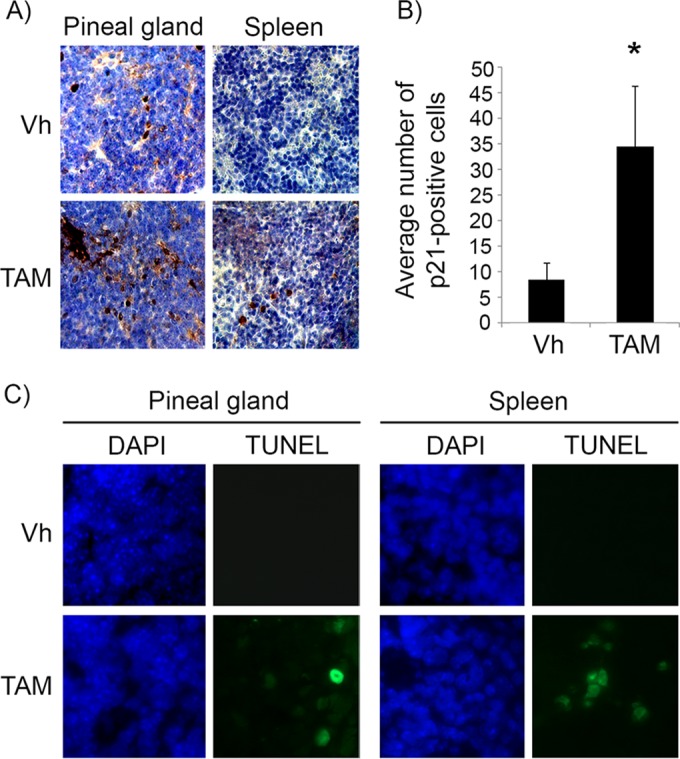
Tamoxifen treatment restores p53 activity in pineal glands *in vivo*. (A) Representative immunostaining for p21^Cip1^ in pineal gland sections and spleen of *Irbp-Cyclin D1*, *p53ER*(*TAM*)^*Ki*/−^ mice that underwent irradiation after treatment for 6 days (P10 to P16) with vehicle (Vh) or tamoxifen (TAM) as indicated. (B) Number of p21-positive cells per field under the conditions shown in panel A. Each point represents the means from 2 independent experiments. Bars represent standard deviations, and an asterisk denotes a statistically significant difference (*P* < 0.05). (C) Representative TUNEL staining (green) and corresponding DAPI nuclear stain (blue) under the same conditions as those for panel A.

In the *Irbp-Cyclin D1* mouse, exogenous cyclin D1 expression in photoreceptor precursor cells results in hyperplasia of the pineal gland that never progresses to invasive tumors because of oncogene-induced senescence (16, 18). In the absence of *p53* (*Irbp-Cyclin D1*, *p53*^−/−^ mice), invasive pineoblastoma develops in 100% of the mice by 3 months of age. We utilized this mouse model to investigate the effects of p53 restoration on premalignant pineal lesions. To do that, we made use of the *p53ER*(*TAM*) knock-in mouse, where the endogenous *p53* gene is fused with a modified estrogen receptor, such that p53 function in cells and tissues is dependent on the provision of the ER(TAM) ligand tamoxifen (10). We first verified that treatment with tamoxifen restores p53 in the mouse pineal gland, similar to what has been shown in other tissues (10). Treatment of 10-day-old mice with tamoxifen indeed restored the p53 response to DNA damage in pineal glands and spleens of *Irbp-Cyclin D1*, *p53ER*(*TAM*)^*Ki*/−^ mice, as shown by an increased expression of the p53 downstream effector p21 and increased apoptosis in response to irradiation (Fig. 1).

**FIG 2:**
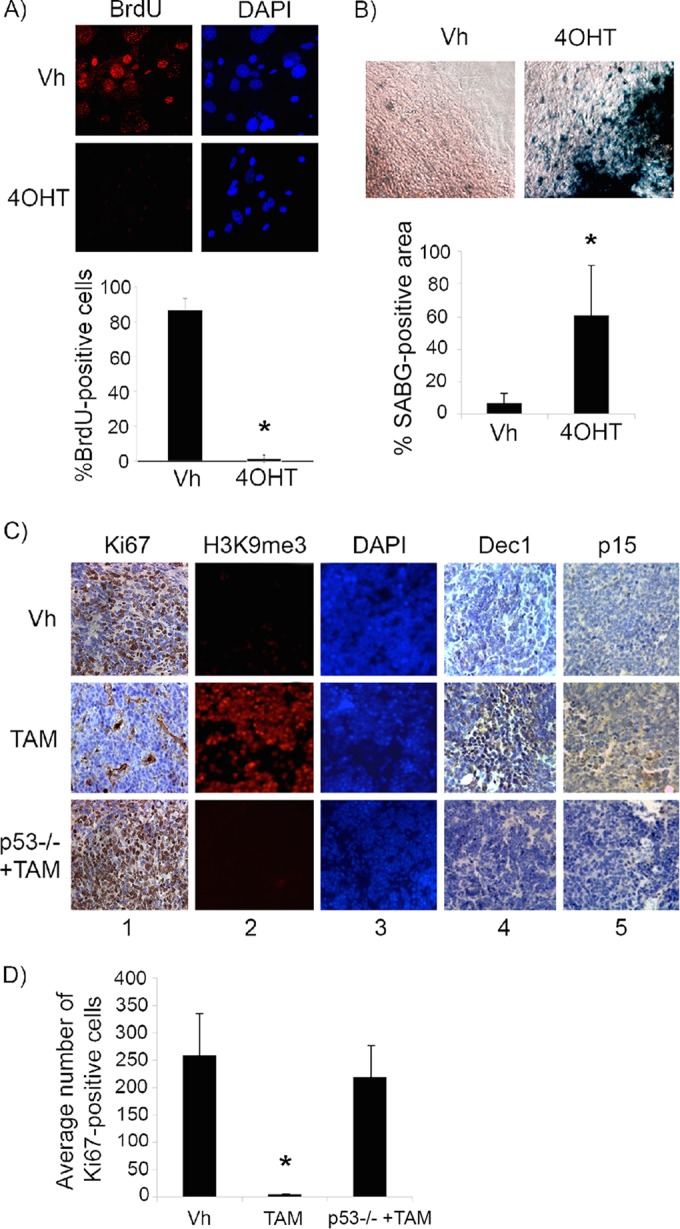
p53 restoration induces senescence in premalignant cyclin D1-expressing pineal cells. (A and B) Shown are representative staining for BrdU along with the corresponding DAPI nuclear stain (A) and senescence-associated beta-galactosidase (SABG) staining (B) in *Irbp-CyclinD1*, *p53ERTAM*^*Ki*/−^ pineal cells explanted at P10 and treated for 7 days with either vehicle (Vh) or 4OHT to restore p53, as indicated. The bottom panels show percent BrdU-positive cells (A) and SABG-positive area (measured in pixel density, as explained in Materials and Methods) (B) under each condition, as indicated. Each point represents the means from 3 independent experiments. (C) Representative immunostaining for the indicated proteins in pineal gland sections from *Irbp-Cyclin D1*, *p53ER*(*TAM*)^*Ki*/−^ mice treated for 10 days (P60 to P70) with Vh or tamoxifen (TAM) and, as controls, *Irbp-Cyclin D1*, *p53*^−/−^ mice treated with TAM (p53−/− + TAM). (D) Number of Ki67-positive cells per field under each condition shown in panel C as indicated. Each point represents the means using at least 4 pineal glands. Bars represent standard deviations, and asterisks denote a statistically significant difference (*P* < 0.05).

We next restored p53 in explanted, premalignant, hyperproliferating P10 *Irbp-Cyclin D1*, *p53ER*(*TAM*)^*Ki*/−^ pineal cells by treatment with 4OHT. Treatment resulted in cell cycle exit (evidenced by decreased BrdU incorporation into DNA) (Fig. 2A) and an increase in senescence-associated beta-galactosidase (SABG) activity (Fig. 2B), a marker of senescence that occurs due to increased lysosome content during cellular senescence (19). We then evaluated whether p53 restoration at a later time point before the development of invasive tumors *in vivo* also would result in the cessation of cell proliferation. At a postnatal age of 60 days (P60), when pineal cell proliferation is continuing and prior to invasive tumor development, which is seen by 3 months of age (18), p53 restoration for a period of 10 days (P60 to P70) resulted in cell cycle exit, as evidenced by the loss of Ki67 positivity (Fig. 2C, column 1, compare top and middle rows, and D). We evaluated these lesions for the expression of markers of senescence, such as senescence-associated heterochromatin foci (SAHF), which are foci of heterochromatin marked by histone 3 trimethylated at lysine 9 (H3K9me3). SAHF are seen in senescent lesions driven by oncogene expression and are thought to contribute to the silencing of cell cycle genes (20). Indeed, tamoxifen-treated pineal glands showed H3K9me3 nuclear foci, consistent with SAHF (Fig. 2C, columns 2 and 3, compare top and middle rows), and also expressed Dec1 and p15Ink4b (Fig. 2C, columns 4 and 5, compare top and middle rows), additional markers of senescence (2, 21, 22).

This effect was due to p53 restoration and not simply to tamoxifen itself, as similar treatment of *Irbp-Cyclin D1*, *p53*^−/−^ animals with tamoxifen did not result in any changes in the above-mentioned markers (Fig. 2C, bottom row, and D). Notably, while p53 restoration by tamoxifen resulted in the cell cycle exit and senescence features, it had no effect on apoptosis (negative data not shown). Thus, we conclude that p53 restoration in premalignant proliferating p53-deficient pineal cells was effective in inducing a senescence-like state, halting further proliferation.

### Restoration of p53 is not effective in suppressing proliferation of invasive tumor cells unless combined with genotoxic stress.

**FIG 3:**
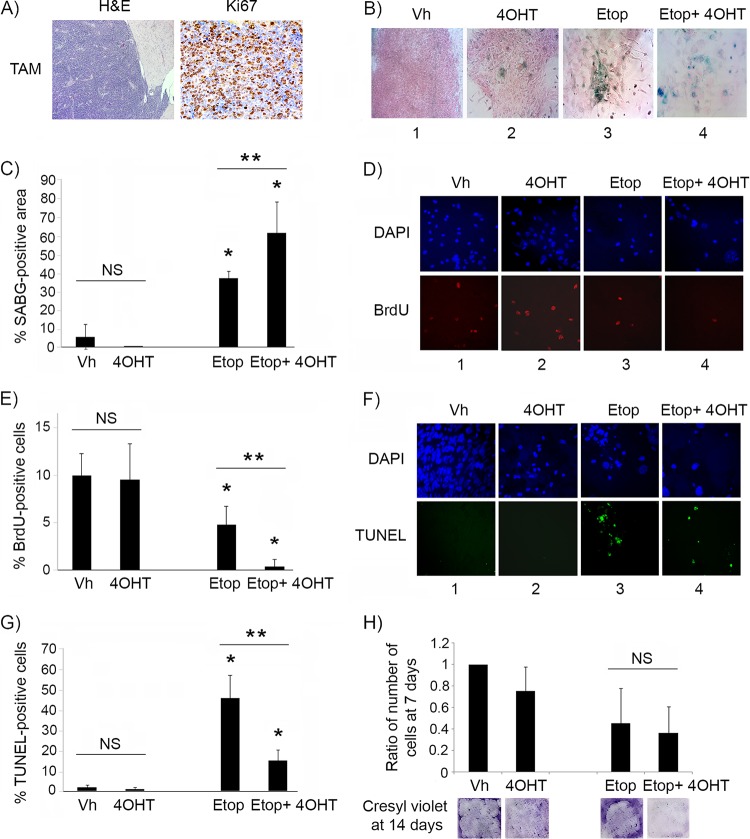
p53 restoration is not effective in suppressing proliferation of malignant pineal tumor cells unless combined with genotoxic stress. (A) Representative hematoxylin and eosin staining (H&E) and Ki67 immunostaining in *Irbp-Cyclin D1*, *p53ER*(*TAM*)^*Ki*/−^ pineal tumors after 6 days of treatment with tamoxifen (TAM) to restore p53. (B) Representative staining for SABG in explanted *Irbp-Cyclin D1*, *p53ERTAM*^*Ki*/−^ pineal tumor cells treated for 7 days with vehicle (Vh), 4OHT to restore p53, etoposide to induce genotoxic stress (Etop), or etoposide and 4OHT (Etop + 4OHT), as indicated. (C) Percent SABG-positive area under the conditions described for panel B, where area was measured in pixel density as explained in Materials and Methods. (D) Representative BrdU level and corresponding DAPI nuclear staining under the same conditions as those for panel B. (E) Percent BrdU-positive cells for the conditions depicted in panel D. (F) Representative TUNEL staining to detect apoptosis, under the same conditions as those for panel B, at 48 h after treatment. (G) Percent TUNEL-positive cells under the same conditions as those for panel F. (H, upper) Quantitation of the total number of cells under the conditions described for panel B at 7 days after treatment and normalized to the vehicle-treated control. (Lower) Cresyl violet stain for colony formation assay under the same conditions at 14 days after treatment. Each point in panels C, E, G, and H represents the means from at least 5 fields and is representative of at least 2 independent experiments. Bars represent standard deviations, asterisks denote a statistically significant difference (*P* < 0.05), and *NS* denotes nonsignificant difference. A single asterisk denotes significance relative to corresponding control conditions (Vh and 4OHT, respectively), while double asterisks denote significance relative to Etop-treated cells, as shown by the horizontal bar.

Unlike the case for animals with premalignant lesions, p53 restoration by tamoxifen treatment of *Irbp-Cyclin D1*, *p53ER*(*TAM*)^*Ki*/−^ mice that had developed visible tumors failed to show any response, and histologic evaluation showed invasive tumors with diffuse Ki67 staining, confirming continued proliferation (Fig. 3A). Similar results were found *in vitro*, as p53 restoration by 4OHT in explanted primary pineal tumor cells had minimal effects on the induction of SABG positivity (Fig. 3B, compare images 1 and 2, and C) or on cell proliferation (Fig. 3D, compare images 1 and 2, and E). We then evaluated whether p53 restoration effects could be augmented by further activation using DNA damage signals. When the explanted tumor cells were exposed to the topoisomerase inhibitor etoposide to induce genotoxic stress, there was a clear response, with an increase in SABG positivity (Fig. 3B, image 3, and C) and a decrease in proliferation (Fig. 3D, column 3, and E). Interestingly, combining etoposide treatment with p53 restoration (using 4OHT) now resulted in a more profound effect on both SABG positivity and cell proliferation (Fig. 3B and D, compare columns 3 and 4, and C and E). Apoptosis also was induced by etoposide treatment in both vehicle- and 4OHT-treated cells (Fig. 3F, compare columns 3 and 4 to columns 1 and 2, respectively, and G), but interestingly p53 restoration seemed protective, reducing the proportion of cells undergoing apoptosis compared to that of cells undergoing etoposide treatment alone (Fig. 3F, compare columns 3 and 4, and G). The number of viable tumor cells 7 days after a single dose of etoposide was similar in cells lacking p53 or with restored p53 (Fig. 3H, upper), suggesting that the effect of p53 restoration on inducing cell cycle exit was quantitatively similar to its protective effect against apoptosis. However, the assessment of cell density by cresyl violet staining after a longer period of 14 days showed that tumor cells treated with etoposide alone seemed to have resumed cell accumulation, whereas those treated with etoposide and receiving p53 restoration (4OHT) had sustained suppression of cell proliferation, consistent with continuous senescence (Fig. 3H, lower).

**FIG 4:**
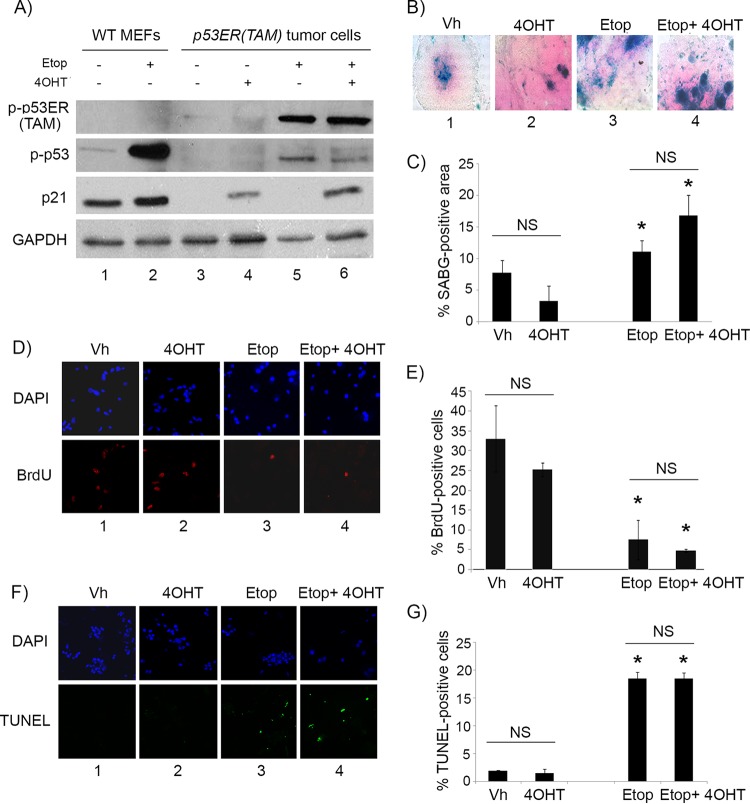
4OHT restores p53 activity in p53ER(TAM) cells and has no effect on explanted *Irbp-Cyclin D1*, *p53*^−/−^ tumor cells. (A) Western blotting for the indicated proteins in wild-type MEFs (WT MEF) as controls and in *p53ERTAM* pineal tumor cells treated for 48 h with 10 μM etoposide (Etop) or vehicle and with 4-hydroxytamoxifen (4OHT) or vehicle. GAPDH was used as a loading control. (B, D, and F) Representative staining for SABG (B) and BrdU (D, lower), and the corresponding DAPI nuclear stain (D, upper), at 7 days after treatment and TUNEL staining (F, lower) and the corresponding DAPI nuclear stain (upper) at 48 h after treatment in explanted *Irbp-Cyclin D1*, *p53*^−/−^ pineal tumor cells treated with vehicle (Vh), 4OHT, etoposide, or both (Etop + 4OHT), as indicated. (C, E, and G) Percentages of SABG-positive area (C), BrdU-positive cells (E), and TUNEL-positive cells (G) under the conditions shown in panels B, D, and F, respectively. Each point represents the means from at least 5 fields and is representative of 2 independent experiments. Bars represent standard deviations, and asterisks denote a statistically significant difference (*P* < 0.05) relative to the respective control conditions (Vh and 4OHT, respectively). NS denotes nonsignificance relative to the condition shown by the horizontal bar.

We verified that 4OHT treatment indeed was restoring p53 activity, as tumor cells showed the induction of protein levels of the p53 downstream effector p21^Cip1^ only under conditions where 4OHT was added (Fig. 4A, compare lanes 4 and 6 to lanes 3 and 5). Etoposide treatment resulted in the phosphorylation of p53 at serine 15/20 (a DNA damage-dependent phosphorylation site) under all conditions, as expected, but with p21^Cip1^ induction only when given concomitantly with 4OHT (Fig. 4A, compare lanes 5 and 6 to lanes 3 and 4). We also verified that the observed effects were not due to 4OHT itself but rather to its effects on p53 restoration by evaluating treatment of explanted *Irbp-Cyclin D1*, *p53*^−/−^ tumor cells. As described above, treatment with 4OHT or vehicle showed no differential effect on SABG positivity (Fig. 4B, compare columns 1 and 2, and C), proliferation (Fig. 4D, compare columns 1 and 2, and E), or apoptosis (Fig. 4F, compare columns 1 and 2, and G). However, unlike in *Irbp-Cyclin D1*, *p53ER*(*TAM*)^*Ki*/−^ tumor cells, the combination of 4OHT with etoposide in *Irbp-Cyclin D1*, *p53*^−/−^ tumor cells resulted in no further effects (Fig. 4B, D, and F, compare columns 3 and 4; quantitation is shown in C, E, and G). Thus, we conclude that, unlike premalignant proliferating lesions where p53 restoration alone induced a senescent phenotype, restoration of p53 in established tumors alone was not efficacious but was able to augment the senescence response while attenuating the apoptotic response to DNA damage-inducing agents such as etoposide.

### p53 is essential for continued maintenance of senescence in premalignant lesions.

**FIG 5:**
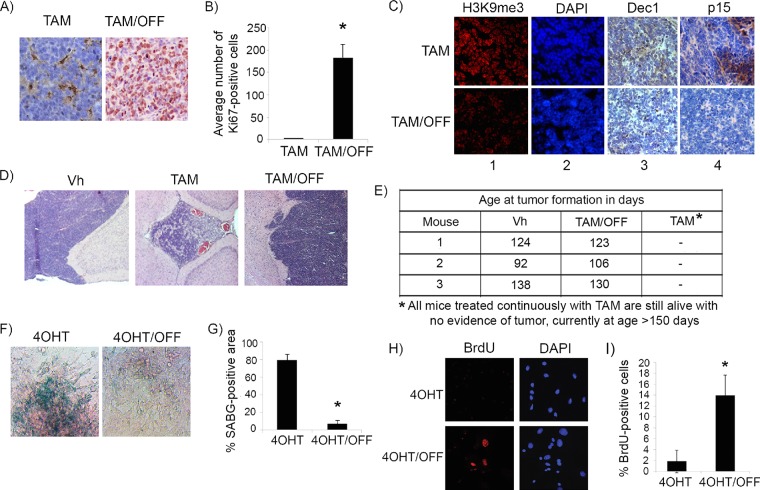
p53 is needed for the maintenance of the senescence-like response in cyclin D1-expressing pineal cells. (A) Representative immunostaining for Ki67 in pineal gland sections of *Irbp-Cyclin D1*, *p53ER*(*TAM*)^*Ki*/−^ mice treated for 10 days (P60 to P70) with TAM to restore p53 and then either treated further with tamoxifen (TAM) or withdrawn from treatment (TAM/OFF) to inactivate p53 for another 10 days, as indicated. (B) Mean number of Ki67-positive cells per field under the conditions shown in panel A. Each point represents the means from at least 5 fields and is representative of 2 independent experiments. (C) Representative immunostaining for the indicated markers of senescence under the same conditions as those for panel A. (D) Representative hematoxylin and eosin staining of *Irbp-Cyclin D1*, *p53ER*(*TAM*)^*Ki*/−^ pineal glands from mice treated for 10 days (P60 to P70) with vehicle (Vh) and from mice that were treated for 10 days with tamoxifen to restore p53 and then either treated further with tamoxifen or that had tamoxifen withdrawn to inactivate p53 (TAM/OFF) for another 10 days, as indicated. (E) Age at clinical tumor formation in a cohort of mice treated with vehicle, tamoxifen for 1 month from P60 to P90 (TAM/OFF), or continuous tamoxifen treatment, as indicated. (F and H) Representative staining for SABG (F) and BrdU (H, left) and corresponding DAPI nuclear stain (H, right) in *Irbp-CyclinD1*, *p53ERTAM*^*Ki*/−^ pineal cells explanted at P10. The cells were treated for 7 days with 4OHT to restore p53 and then either treated further with 4OHT (4OHT) or withdrawn from tamoxifen treatment to inactivate p53 (4OHT/OFF) for another 7 days as indicated. (G and I) Percentages of SABG-positive area (G) and BrdU-positive cells (I), under the conditions shown in panel F and H, respectively, are shown. Each point represents the means from at least 5 fields and is representative of 2 independent experiments. Bars represent standard deviations, and asterisks denote a statistically significant difference (*P* < 0.05).

To evaluate the stability of p53-mediated tumor suppression, we treated *Irbp-Cyclin D1*, *p53*^*Ki*/−^ mice at P60, as described above, for 10 days with tamoxifen. The mice then either continued to receive tamoxifen (continued p53 restoration) or were treated with vehicle only (tamoxifen withdrawal to inactivate p53) for 10 more days. Inactivation of p53 for 10 days resulted in the reversal of the senescence phenotype, with massive resumption of cell proliferation evidenced by positive staining for Ki67 (Fig. 5A and B) and a decrease in markers of senescence, such as SAHF, Dec1, and p15^Ink4b^ (Fig. 5C, compare upper and lower rows). Of note, this resumption of proliferation was clinically significant, because lesions with continued tamoxifen treatment were localized with no evidence of brain tissue invasion (Fig. 5D, middle), while those with tamoxifen withdrawal showed tumor invasion into brain parenchyma (Fig. 5D, right), similar to vehicle-treated lesions where p53 was never restored (Fig. 5D, left). Clinically, a cohort of mice treated continuously since age P60 with tamoxifen showed no evidence of tumor at ages of >150 days, whereas vehicle-treated littermates all succumbed to tumors that manifested as skull protrusions and ill appearance at ages of 92 to 138 days. On the other hand, a cohort of mice treated with tamoxifen from P60 to P90, after which tamoxifen was stopped, all developed clinically apparent tumors within 16 to 40 days after tamoxifen withdrawal (Fig. 5E). We conclude that cells that reentered the cell cycle upon loss of p53 function were able to complete cell division and rapidly acquire invasive properties.

To evaluate whether the reversion of senescence after p53 inactivation was influenced by the prolonged proliferation time that preceded p53 restoration *in vivo*, we evaluated young (P10) pineal cells for the effect of p53 inactivation after senescence. Cell culture explants of pineal cells from 10-day-old *Irbp-Cyclin D1*, *p53ER*(*TAM*)^*Ki*/−^ mice were allowed to undergo senescence by p53 restoration (4OHT) treatment *in vitro* for 1 week, as described previously (Fig. 2A and B). 4OHT then was withdrawn, and cells were evaluated 1 week later. Similar to our results *in vivo* with older mice, we found that p53 inactivation effectively reverted the senescent phenotype, with the loss of SABG positivity (Fig. 5F and G) and the resumption of cell proliferation, as shown by increased incorporation of BrdU into DNA (Fig. 5H and I). Thus, we conclude that p53 is needed for maintaining the senescent state and effectively halting proliferation.

### p53 also is necessary for maintenance of Ras^V12^-induced senescence in mouse fibroblasts.

**FIG 6:**
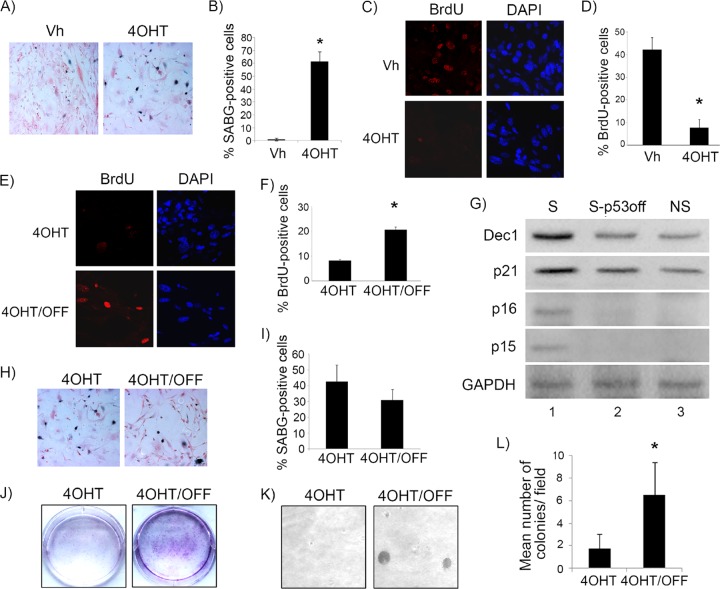
p53 is necessary for maintenance of RasV12-induced senescence in fibroblasts. (A) Representative staining for SABG in *p53ERTAM*^*Ki*/−^ MEFs transduced with *RasV12* and treated with either vehicle (Vh) or 4-OH tamoxifen (4OHT) to restore p53, as indicated. (B) Percent SABG-positive cells under the conditions represented in panel A. (C) BrdU and corresponding DAPI nuclear stain in *p53ERTAM*^*Ki*/−^ MEFs treated as described for panel A. (D) Percent BrdU-positive cells under the conditions represented in panel C. (E) Representative staining for BrdU and corresponding DAPI nuclear stain in *p53ERTAM*^*Ki*/−^ MEFs after *RasV12* transduction and treatment with 4OHT to restore p53 for 1 week. After this, 4OHT treatment continued (4OHT) or was withdrawn to inactivate p53 (4OHT/OFF) for another week. (F) Percent BrdU-positive cells under the conditions represented in panel E. (G) Western blotting for the indicated proteins in *p53ERTAM*^*Ki*/−^ MEFs after *RasV12* transduction and treatment with 4OHT to restore p53 for 2 weeks (senescent cells [S]) or treatment with 4OHT for 1 week and then withdrawal to inactivate p53 for another week (S-p53OFF) or in control, *RasV12*-transduced, vehicle-treated MEFs as never-senescent controls (NS). (H) Representative staining for SABG in *p53ERTAM*^*Ki*/−^ MEFs treated as described for panel E. (I) Percent SABG-positive cells under the conditions represented in panel H. (J and K) Cell density assay by Cresyl violet staining (J) and soft-agar colony formation assay (K) in *RasV12*-transduced *p53ERTAM*^*Ki*/−^ MEFs after treatment with 4OHT to restore p53 for 1 week. Treatment was continued (4OHT) or was withdrawn to inactivate p53 (4OHT/OFF) for another 2 weeks, as indicated. (L) Mean number of colonies per field under each condition shown in panel K. Each point in panels B, D, F, I, and L represents the means from at least 5 fields and is representative of at least 2 independent experiments. Bars represent standard deviations, and asterisks denote a statistically significant difference (*P* < 0.05).

To evaluate the role of p53 in the maintenance of oncogene-induced senescence in other settings, we made use of the well-established model of Ras^V12^-induced senescence in mouse embryo fibroblasts (MEFs), which depend on active p53 for the induction of senescence (23, 24). To be able to inactivate *p53* after the onset of senescence, we again used the *p53ER*(*TAM*)^*Ki*/−^ model discussed above. We first verified that, as expected, the induction of senescence by oncogenic Ras^V12^ is dependent on *p53* in the mouse fibroblasts, such that a senescent phenotype is seen in *RasV12*-transduced, *p53ER*(*TAM*)^*Ki*/−^ MEFs only when they are treated with 4OHT, as shown by the induction of SABG positivity (Fig. 6A and B), and cell cycle exit, as evidenced by the loss of BrdU incorporation (Fig. 6C and D). Similar to cyclin D1-driven senescence in mouse pineal cells, we found that the inactivation of p53 after senescence (at day 14 after *RasV12* transduction) resulted in the reversion of the senescence phenotype, with the resumption of DNA synthesis evidenced by an increase in BrdU incorporation into DNA (Fig. 6E and F) and a decrease in the expression of the senescence markers Dec1, Cdkn1a (p21^Cip1^), p16^Ink4a^, and p15^Ink4b^ (Fig. 6G, compare lane 2 to lane 1). While there seemed to be some decrease in SABG-positive cells at this time point, it was not statistically significant (Fig. 6H and I), showing that this particular marker of senescence persisted at this stage in the majority of cells despite restored DNA synthesis.

Importantly, progressive cell accumulation occurred, in contrast to that of cells with continued p53 restoration (by continued 4OHT treatment) (Fig. 6J), as did increased colony formation in soft agar (Fig. 6K and L), demonstrating successful reversion of senescence and completion of proliferation and subsequent cell divisions after p53 inactivation.

### Efficacy of p53 restoration *in vivo* is not related to status of its upstream activator p19^Arf^ but is inversely correlated with Mdm2 expression.

**FIG 7:**
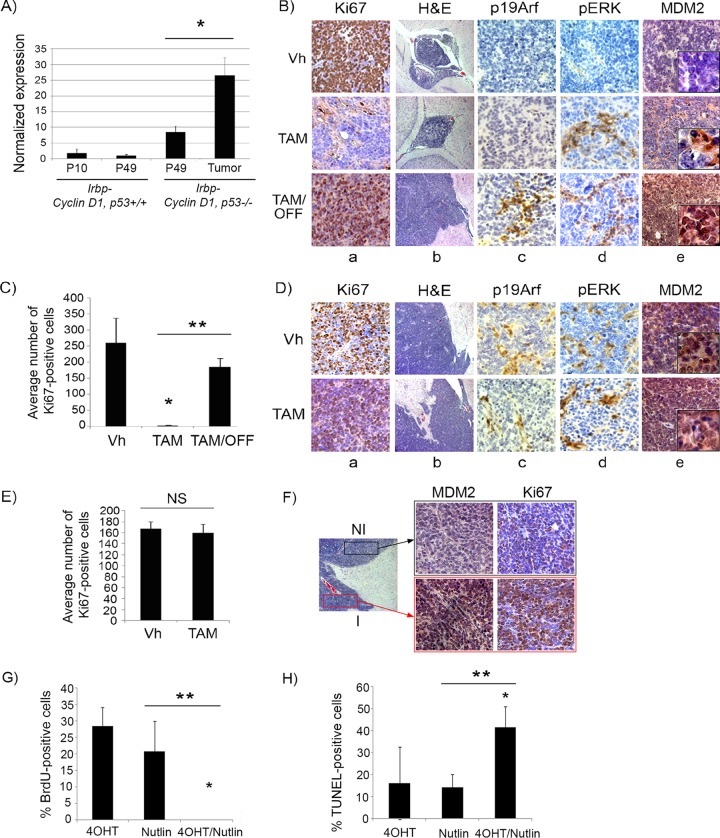
Mdm2 but not p19^Arf^ expression levels correlate with p53 restoration efficacy in pineal tumors. (A) qRT-PCR analysis of mRNA expression levels of *p19Arf* relative to those of *GAPDH* in *Irbp-Cyclin D1*, *p53*^*+/+*^ pineal glands at P10 (proliferating, presenescent) or P49 (senescent) and *Irbp-Cyclin D1*, *p53*^−/−^ pineal glands at the pretumorigenic (P49) or tumor (Tumor) stage, as indicated. Each point represents the means from 3 independent experiments. Bars represent standard deviations, and asterisks denote a statistically significant difference (*P* < 0.05). (B) Representative immunostaining for the indicated proteins and hematoxylin and eosin staining (H&E) in *Irbp-Cyclin D1*, *p53ER*(*TAM*)^*Ki*/−^ premalignant lesions treated with vehicle (Vh), tamoxifen from P60 to P80 (TAM), and tamoxifen for 10 days from P60 to P70 followed by its withdrawal from P70 to P80 (TAM/OFF). The inset in column e shows a magnified image. (C) Average number of Ki67-positive cells per field under the conditions described for panel B. (D) Representative immunostaining for the indicated proteins and H&E staining in *Irbp-Cyclin D1*, *p53ER*(*TAM*)^*Ki*/−^ pineal invasive tumors treated with vehicle (Vh) or tamoxifen for 10 days (TAM). The inset in column e shows a magnified image. (E) Average number of Ki67-positive cells per field under the conditions described for panel D. Each point in panels C and E represents the means from at least 4 different mouse pineal sections. Bars represent standard deviations. A single asterisk denotes significance (*P* < 0.05) relative to corresponding control conditions (Vh), while double asterisks denote significance relative to results for TAM-treated mice, as shown by the horizontal bar. NS denotes nonsignificance. (F) Immunostaining for MDM2 (left) and Ki67 (right) in an *Irbp-Cyclin D1*, *p53ER*(*TAM*)^*Ki*/−^ pineal lesion in transformation that has both premalignant noninvasive (NI) and invasive (I) tumor components. (G) Percent BrdU-positive cells in explanted *Irbp-Cyclin D1*, *p53ERTAM*^*Ki*/−^ pineal tumor cells 7 days after treatment with 4OHT, nutlin, or both, as indicated. (H) Percent TUNEL-positive cells after 24 h of treatment under the same conditions as those for panel G. Each point in panels G and H represents the means from at least 5 different fields. Bars represent standard deviations. A single asterisk denotes significance (*P* < 0.05) relative to the 4OHT control condition, while double asterisks denote significance relative to the nutlin condition, as shown by the horizontal bar.

Oncogenic signaling to p53 occurs in most instances through the induction of *p19Arf* (*p14ARF* in humans), which then leads to p53 stabilization by binding and inactivating MDM2 (HDM2 in humans), a negative regulator of p53 (25). Several other models of oncogene-induced senescence have found *Arf* to be an upstream regulator of p53 activation and senescence induction (26), and previous studies have shown that the efficacy of p53 restoration depends on levels of p19^Arf^, such that lesions with high p19^Arf^ expression show responses to p53 restoration and vice versa (14, 15, 27). We evaluated whether *p19Arf* status could explain the differential effects of p53 restoration in premalignant and malignant tumors in our model. Consistent with our previous findings where *p19Arf* was found to be dispensable for p53-mediated cellular senescence in this model (18), we found that the *p19Arf* transcript levels did not increase during senescence of *Irbp-Cyclin D1* pineal cells, as levels at P10 (proliferating presenescent cells) and P49 (senescent cells) were similar (Fig. 7A). *p19Arf* was expressed to higher levels in the proliferating premalignant lesions (*Irbp-Cyclin D1*, *p53*^−/−^ pineal glands at P49) than in senescent age-matched pineal glands from *Irbp-Cyclin D1*, *p53* ^*+/+*^ mice, and it was expressed to even higher levels in *Irbp-Cyclin D1*, *p53*^−/−^ tumors (Fig. 7A). In premalignant hyperproliferating pineal lesions, in which p53 restoration results in cell cycle exit (Fig. 7B, columns a and b, compare top and middle rows, and C), we found low p19^Arf^ expression on immunostaining (Fig. 7B, top row, column c). Interestingly, reversion from senescence after tamoxifen withdrawal now resulted in both resumed proliferation (Fig. 7B, columns a and b, bottom row, and C) and increased expression of p19^Arf^ (Fig. 7B, column c, bottom row). On the other hand, invasive malignant tumors, which do not respond to p53 restoration (Fig. 7D, columns a and b, compare upper and lower rows, and E), were positive for p19^Arf^ expression (Fig. 7D, column c). Thus, from the preceding data, we conclude that p19^Arf^ positivity did not predict the response to p53 restoration in this model.

Since Arf acts primarily by inhibiting the p53-Mdm2 interaction (28–30), we considered that alterations in Mdm2 may be responsible for the inhibition of effective p53 activation. The amplification of *Mdm2* and/or the upregulation of its protein expression have been shown to be responsible for p53 inactivation (31–35) and are seen in multiple tumor types (36–39). Indeed, by immunostaining for Mdm2, we found that levels were low in premalignant lesions and elevated in malignant tumors (Fig. 7B and D, top rows, column e), correlating with p53 response. Consistent with this, the evaluation of an early pineal tumor where part of the lesion showed invasive features (lesion in transformation) revealed that the portions of the lesion that were invading surrounding tissue had high levels of Mdm2 protein expression, while the noninvasive, premalignant portion of the lesion showed low-level staining (Fig. 7F). Indeed, treatment of explanted pineal tumor cells with nutlin, a small-molecule inhibitor of the p53-mdm2 interaction (40), resulted in effective cell cycle exit and apoptosis upon p53 restoration by 4OHT treatment (Fig. 7G and H), proving the importance of the p53-Mdm2 interaction in abrogating p53 activity in this setting.

### Restoration of p53 leads to upregulation of oncogenic signaling and induction of p19^Arf^.

Since p53 activation has been shown previously to upregulate the mitogen-activated protein kinase (MAPK) pathway by a feedback loop in malignant cells (41–44), we assessed whether the level of oncogenic signaling, measured by MAPK pathway activation, was affected by p53 restoration. We found that phosphorylated ERK (pERK), a downstream effector of MAPK signaling, was not detectable in proliferating premalignant lesions (Fig. 7B, top row, column d), whereas, similar to p19^Arf^, foci of pERK positivity were evident in malignant tumors (Fig. 7D, upper row, column d). Once p53 was restored by tamoxifen treatment, foci of pERK positivity appeared in the arrested premalignant lesions (Fig. 7B, middle row) and continued to be expressed after tamoxifen withdrawal, when lesions started proliferating again (Fig. 7B, bottom row). This correlation between pERK and p19^Arf^ positivity in proliferating lesions is in concordance with previous findings that p19^Arf^ expression responds to a threshold of upstream oncogenic signaling (15). Therefore, on the basis of the above-described data, we propose that the restoration of p53 leads not only to senescence but also to the augmentation of upstream oncogenic signaling and secondary p19^Arf^ expression. When p53 subsequently is inactivated, enhanced oncogenic signaling is maintained, likely contributing to the observed rapid tumor progression (Fig. 7B, column b, compare bottom image to top two images, and 5E).

### Restoration of the p53 pathway may be a suitable target in human sPNET.

**FIG 8:**
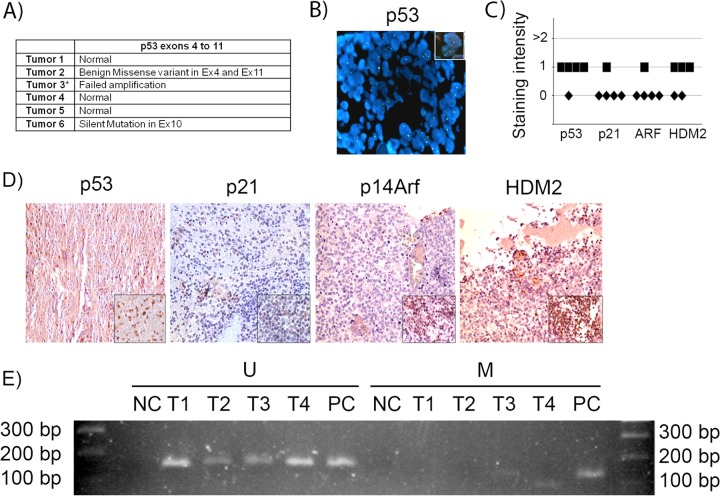
p53 gene and pathway likely are not active in human sPNET. (A) Results of sequencing of *p53* exons 4 to 11 in 6 human sPNET samples. (B) FISH staining for *p53* gene deletion in tumor 3. The inset shows a positive control. (C) Quantitation of the intensity of expression of p53, p21^CIP1^, p14^ARF^, and HDM2, detected by immunohistochemistry, in sPNET samples. (D) Representative images of immunostaining for the indicated proteins in human sPNET samples. The insets represent the respective positive controls. (E) Analysis of p14^Arf^ promoter methylation status in sPNET samples (T1, T2, T3, and T4), showing unmethylated (U) and methylated (M) sequences. NC, negative control; PC, positive control.

We evaluated 6 human samples of human supratentorial primitive neuroectodermal tumors (sPNET) (of which pineoblastoma is a subtype) for p53 gene mutations and deletions and for evidence of the deregulation of the p53 pathway. The sequencing of exons 4 to 11 revealed no deleterious mutations in the *p53* gene in 5 of 6 tumors, while amplification was not successful in one tumor, suggesting deletion (Fig. 8A). Further analysis of that tumor by FISH showed that the p53 gene was indeed deleted (Fig. 8B). By immunostaining, the level of expression of both p53 and its downstream effector, p21^Cip1^, were either absent or low in all tumor samples (Fig. 8C and D). Immunostaining for HDM2 (the human homologue of MDM2), which may be amplified in tumors and, in such cases, acts to ubiquitylate the p53 protein and enhance its degradation (45, 46), showed low expression in all tested tumor samples (Fig. 8C and D). Immunostaining for p14^Arf^ (the human homologue of p19^Arf^) showed that ARF was not expressed or had very low levels of expression in all examined samples (Fig. 8C and D). To examine whether the p14ARF promoter was methylated, which is a process by which *ARF* is commonly repressed in other tumors (47), we conducted methylation-specific PCR of the promoter sequence. Bisulfite treatment of DNA converts unmethylated cytosine residues into uracil, but methylated cytosine residues remain unmodified, allowing the distinction of methylated and unmethylated DNA sequences by sequence-specific PCR primers. We found that *p14ARF* promoter methylation was absent from most tumors (Fig. 8E). Therefore, we conclude that the p53 pathway is inactive in sPNET even in the large subset of cases where the p53 gene is intact, with the lack of p14^ARF^ activation and also the lack of its repression by promoter methylation, at least in the samples we tested.

## DISCUSSION

We found that p53 restoration was effective in preventing tumor progression in premalignant proliferating murine pineal lesions, even after prolonged periods of proliferation, by driving cells into a senescence-like state. On the other hand, simple p53 restoration in established invasive tumor cells was ineffective unless it was coupled with DNA-damaging therapy (etoposide), suggesting that the signaling pathway linking oncogenic stimuli to p53 activation was deficient in this setting. Thus, at least in cyclin D1-driven pineoblastoma, the restoration of p53 alone may be sufficient to control the progression of premalignant proliferating lesions, demonstrating a proof of principle for a prevention approach that may be especially desirable for patients with high-risk premalignant conditions.

In our model, the effectiveness of p53 restoration correlated with the status of Mdm2 positivity, with advanced tumors (that did not respond to simple p53 restoration) having higher expression of Mdm2 than premalignant tumors (which did respond to simple p53 restoration). Responsiveness to p53 was reestablished after treatment with nutlin, a small-molecule inhibitor of the p53-mdm2 interaction, consistent with the lack of effectiveness being due to Mdm2-mediated inactivation of the restored p53 in tumor cells. p53 restoration previously has been shown to be effective in other tumor models, such as in *HRasV12*-driven liver tumors, where it results in tumor cell senescence and subsequent clearance (48), and in primary tumors of p53-deficient mice, where it leads to rapid apoptosis in lymphomas and tumor cell senescence in sarcomas (49). In HRas^V12^-driven glioma and Eu-Myc-driven lymphoma, p53 restoration in tumors was effective but depended primarily on the presence of p19^Arf^ upstream signaling to p53 (14, 50), and the restoration of p53 in KRas-driven lung tumors led to responses in malignant carcinomas but not in adenomas (15, 27), also in a manner dependent on p19^Arf^.

Thus, both our study and those of others (14, 15, 27, 50) show that the restoration of p53 in advanced tumors where upstream oncogenic signaling to p53 is impaired still may be effective when combined with DNA-damaging therapies. In both cases, the effect correlates with the status of the Arf/Mdm2 axis, with the only divergence being that regarding the specific role of p19^Arf^ (in the studies referenced above) versus Mdm2 (in our study) in modulating the efficacy of p53 restoration. Notably, Mdm2 recently has been found to be subject to multiple p53-independent regulators (reviewed in references 51 and 52) and to exhibit p53-independent oncogenic activity, with tumors showing both Mdm2 amplification and p53 loss/mutation faring especially poorly (reviewed in references 52 and 53). Thus, our findings, in the context of previous studies, verify the importance of evaluating the status of both Arf and Mdm2 when considering the possible efficacy of p53 pathway restoration in tumor cells.

Interestingly, while p53 restoration combined with DNA-damaging therapy (etoposide) in pineal tumor cells resulted in an enhanced effect on senescence induction, it also had a protective effect against apoptosis. Other studies recently also have shown a protective effect of wild-type p53 on therapy-induced apoptosis. In breast cancer induced by *MMTV-Wnt1* in mice and in human breast cancer cell lines *in vitro*, it was shown that doxorubicin treatment results in senescence induction in *p53* wild-type tumors and in apoptosis in *p53*-deficient tumors (54). In that study, there was significantly faster tumor regrowth in mice with *p53* wild-type tumors than in mice with *p53*-deficient tumors. Similarly, another study showed that functional p53 signaling was associated with chemoresistance (55). In our study, the analysis of surviving cells 7 days after etoposide treatment showed no difference under conditions with restored p53 activity or lacking p53 activity, suggesting that the respective effects on senescence and apoptosis were similar. Further analysis 14 days after etoposide treatment showed that the protection against apoptosis in cells with restored p53 did not translate into a growth advantage; on the contrary, cells with restored p53 had persistent growth arrest, whereas those remaining after treatment with etoposide alone had resumed cell accumulation. Our study was different from those mentioned above in several features, such as cell type (pineal tumor cells versus breast cancer cells) and primary tumor cells (in our model) versus established cancer cell lines.

Another interesting finding in our study was that levels of p19^Arf^ expression were low in proliferating premalignant lesions but high in invasive tumors. Of note, p19^Arf^ induction has been shown to depend on the level of oncogenic signaling, such that a threshold of such signaling is needed to engage Arf expression during the transition from premalignant to malignant tumors (56, 57). Therefore, we propose that the absence of p19^Arf^ in premalignant pineal lesions in this model can be explained by a low level of oncogenic signaling below the threshold needed to be sensed by p19^Arf^, although it was high enough to cause continued cellular proliferation (39, 40). In invasive tumors, the increased level of oncogenic signaling (evidenced by increased pERK) now was sufficient to induce *p19Arf* but was ineffective for tumor suppression in the absence of p53. Interestingly, p19^Arf^ expression was seen in premalignant tumors only after p53 restoration and secondary reversion from senescence after subsequent p53 inactivation. This was accompanied by MAPK pathway activation evidenced by pERK expression. This finding can be explained by recent studies showing that p53 activation can lead to the secondary upregulation of the MAPK pathway through mechanisms that include the p53-dependent generation of reactive oxygen species (43, 44) and p53-dependent upregulation of Thrombospondin-1, which directly interacts with pERK to increase MAPK signaling (42). Thus, it is plausible that, in premalignant lesions, p53 restoration led to both cellular senescence and secondary activation of the MAPK pathway, similar to what was reported previously (42–44), and this then led to an oncogenic signaling threshold stimulating p19^Arf^ expression. As evidenced by continued pERK expression, such heightened oncogenic signaling continued after p53 inactivation by tamoxifen withdrawal, now leading to reentry into the cell cycle, reversion to senescence, and the observed secondary rapid tumor progression.

If p53 activation were to be used as a therapeutic approach, it would be imperative to know whether such therapy would result in irreversible tumor cell control or whether p53 activation would need to be continually maintained for stable tumor suppression. Our data suggest the latter, as the maintenance of the senescence-like state was dependent on intact p53. This was true for cyclin D1-expressing pineal cells and Ras^V12^-expressing fibroblasts, where the loss of p53 promoted successful renewed cell proliferation and colony formation. In Ras^V12^-induced senescence in MEFs, we noted that after p53 inactivation, there was no difference in SABG positivity despite the resumption of cell proliferation. This was different from senescence reversion in cyclin D1-expressing pineal cells, where SABG positivity decreased in concordance with increased proliferation. These differences might be attributable to the different cell types (pineal cells versus fibroblasts), oncogenic signal (cyclin D1 versus Ras), or passage levels (primary pineal cells versus established MEF cell line). Regardless, the fact that cells reentered the cell cycle and completed cell division, resulting in increased cell accumulation and colony formation on soft agar, clearly demonstrates the reversion of senescence despite the residual SABG positivity. Similarly, *in vivo*, p53 inactivation in senescent premalignant lesions driven by cyclin D1 led to rapid reentry into the cell cycle and progression into invasive tumor production. This identifies p53 as an essential effector of senescence maintenance in tumor suppression and reinforces the need for persistently intact p53 activity for the prevention of tumor progression in premalignant and malignant cells.

To the best of our knowledge, ours is the first report of senescence reversion *in vivo*. In replicative senescence, there have been reports of senescence reversion *in vitro* after p53 inactivation (7, 9, 58) and in oncogene-induced senescence *in vitro* in MEFs (10). Notably, in replicative senescence in human fibroblasts, the inactivation of p53 leads to cell cycle reentry, but then cells go into crisis and undergo cell death, with the failure of successful division and ensuing proliferation (7). The question of whether the same happens during the reversion of oncogene-induced senescence in human cells will need to be investigated further, preferably using both human epithelial and mesenchymal cell types and both *in vitro* and *in vivo* (xenograft) models.

Our model of cyclin D1-driven pineoblastoma most closely resembles human sPNET, where cyclin D/CDK amplifications are frequent (59). Pineoblastoma is histologically indistinguishable from other sPNET but is named separately based on location and putative cell of origin (60, 61). Both pineoblastoma and other sPNET are aggressive tumors that have a poor outcome despite multimodality therapy (62, 63). Very few studies have evaluated the molecular pathways underlying these relatively rare tumors, and current work is under way to identify targets for novel therapies, which are much needed (61). The p53 pathway likely is important in sPNET tumor suppression, since most mouse models of sPNET require the inactivation of both the p53 and RB pathways (64).

Our evaluation of 6 sPNET samples showed that the p53 gene is intact in 5 out of 6 tumors, similar to a previous study which also showed that p53 mutations are uncommon in this tumor (65). In addition, we found low expression of the p53 target protein p21^Cip1^. While p53 target genes normally are not expressed in tissues that are wild type for p53, one would expect the activation of this pathway in p53 wild-type tumor cells, in view of the expected ongoing oncogenic signaling in tumors. Our data suggest that while p53 is wild type, it is not effectively activated in tumor cells, contrary to expectations. This correlates with the observed low ARF expression, which would result in an effect similar to that of Mdm2 upregulation (seen in murine tumors), as Arf primarily acts by binding Mdm2 to prevent its effect on p53 inactivation (28–30). Therefore, based on this and our mouse model findings, we suggest that p53 restoration therapies may be efficacious in sPNET. Importantly, several approaches utilizing p53 restoration in humans have started entering preclinical and clinical trials. These include agents that stabilize p53 by inhibiting the MDM2-p53 interaction, such as nutlins and other small-molecule and peptide inhibitors (66–71), and p53 gene therapy in tumors that have lost p53 (70, 72, 73). Combined therapy with DNA-damaging agents (such as radiation or chemotherapeutic agents), coupled with an assessment of the upstream ARF/HDM2 signaling pathway, is likely to better enable p53 activation in tumors that have an intact p53 gene.
